# Methodological Variations Contributing to Heterogenous Ergogenic Responses to Ischemic Preconditioning

**DOI:** 10.3389/fphys.2021.656980

**Published:** 2021-04-29

**Authors:** Liam O'Brien, Ira Jacobs

**Affiliations:** Human Physiology Laboratory, Faculty of Kinesiology and Physical Education, University of Toronto, Toronto, ON, Canada

**Keywords:** ischemic preconditioning, exercise, ergogenic aid, IPC exercise, athletic performance, aerobic exercise, anaerobic exercise, athlete

## Abstract

Ischemic preconditioning (IPC) has been repeatedly reported to augment maximal exercise performance over a range of exercise durations and modalities. However, an examination of the relevant literature indicates that the reproducibility and robustness of ergogenic responses to this technique are variable, confounding expectations about the magnitude of its effects. Considerable variability among study methodologies may contribute to the equivocal responses to IPC. This review focuses on the wide range of methodologies used in IPC research, and how such variability likely confounds interpretation of the interactions of IPC and exercise. Several avenues are recommended to improve IPC methodological consistency, which should facilitate a future consensus about optimizing the IPC protocol, including due consideration of factors such as: location of the stimulus, the time between treatment and exercise, individualized tourniquet pressures and standardized tourniquet physical characteristics, and the incorporation of proper placebo treatments into future study designs.

## Introduction

The induction of transient cycles of arterial blood flow occlusion and reperfusion confers beneficial effects on tissues undergoing subsequent ischemic insults. This technique, known as ischemic preconditioning (IPC), has been shown to improve tissue tolerance to subsequent ischemia in tissues subject to the ischemic intervention (Murry et al., 1986; Dickson et al., 2000), and also remotely located tissues (Kristiansen et al., 2005; Shimizu et al., 2007). Remote IPC (RIPC) is known to promote cardioprotective (Konstantinov et al., 2005; Kristiansen et al., 2005), and neuroprotective effects against infarction in animal models (Pérez-Pinzón, 2004; Liu et al., 2016; Wang et al., 2016). However, evidence of such protection in humans is equivocal.

Perhaps it is this strong evidence of improved local and systemic tissue protection found in animal models that led to the interest of exercise scientists in IPC as a potential ergogenic aid for improving athletic performance. That interest has resulted in several reports demonstrating that both IPC and RIPC can confer positive effects on exercise performance over a variety of exercise intensities, durations, and modalities (Jean-St-Michel et al., 2011; Paradis-Deschênes et al., 2016; Paull and Van Guilder, 2019). But not all studies have reported ergogenic effects with IPC interventions (For review on the ergogenic responses to IPC see: Marocolo et al., 2015b, 2019; Incognito et al., 2016; Salvador et al., 2016; Caru et al., 2019b). Incognito et al. (2016) reported positive IPC response rates of 70 and 74% during time trials of predominantly aerobic and glycolytic anaerobic capacity, respectively. Marocolo et al. (2015b) reported that out of 20 high-quality IPC exercise studies, the majority did not report significant changes to athletic performance or related physiological mediators of performance following IPC interventions. In contrast, and more recently, Caru et al. (2019b) reviewed 52 high-quality articles and found that most studies demonstrated beneficial effects on performance. Our interpretation of these different perspectives is that the influence of IPC on exercise and athletic performance remains equivocal. Moreover, the challenges of publishing negative findings may have further obfuscated the literature on the efficacy of IPC on exercise performance.

In this review we propose that one of the reasons for the equivocal findings may be the widely varying methodological approaches to IPC interventions reported in the literature (Table 1). The primary objective of the current review is, therefore, to describe and consider these variations. Table 1 has been constructed to succinctly describe key aspects of the IPC methodologies used in the IPC exercise studies reported in English language literature to date.

**Table 1:** Methodological summary of studies examining IPC on exercise performance variables.

					**Treatment**				
**References**	**Exercise**	**Type of IPC**	**Location of IPC**	**Protocol**	**IPC**	**CON**	**PLA**	**Method to confirm AOP**	**Time to exercise**
Andreas et al. (2011)	Isometric plantarflexion MVC	Acute IPC	Right thigh (unilateral)	3 × 5 min + 10 min reperfusion	200 mm Hg	Reference test	None	NA	Immediately
Arriel et al. (2018)	Incremental exercise test (Cycling)	Acute IPC PE	Alternate thighs (unilateral)	2 × 5 min 5 × 2 min	50 mm Hg > SBP	None	20 mm Hg	Auscultation	24 h
Arriel et al. (2020)	Incremental exercise test (Cycling)	Acute IPC	Alternate thighs (unilateral)	2 × 5 min	50 mm Hg > SBP	No occlusion	20 mm Hg	NA	NA
Baikoglu and Kaldirimci (2019)	Wingate Anaerobic Test	Acute IPC	Thigh (bilateral)	1 × 5 min	NA	Reference test	None	NA	Immediately
Bailey et al. (2012)	Incremental exercise test + 5 km TT (Running)	Acute IPC	Thigh (bilateral)	4 × 5 min	220 mm Hg	20 mm Hg	None	NA	Immediately
Banks et al. (2016)	Incremental exercise test (Cycling)	Repeat RIPC	Right arm (unilateral)	4 × 5 min for 9 days	200 mm Hg	Reference test	None	NA	24 h
Barbosa et al. (2015)	Rhythmic handgrip TTE at 45% MVC	Acute	Thigh (bilateral)	3 × 5 min	200 mm Hg	10 mm Hg	None	Doppler	25 min
Beaven et al. (2012)	Lower body strength/power testing + repeat sprint (Running)	Acute	Alternate thighs (unilateral)	2 × 3 min	220 mm Hg	15 mm Hg	None	NA	Immediately
Behrens et al. (2020)	Isometric knee extension TTE at 20% MVC	Acute	IPC (legs)	3 × 5 min	120% AOP	None	20 mm Hg	Doppler	20 min
Caru et al. (2016)	Steady state test at 75% and 115% GET (Cycling)	Acute RIPC	Right arm (unilateral)	4 × 5 min	50 mm Hg > SBP	10 mm Hg	None	Manual palpation	5 min
Caru et al. (2019a)	Steady state test at 75% and 115% GET (Cycling)	Acute RIPC	Right arm (unilateral)	4 × 5 min	50 mm Hg > SBP	10 mm Hg	None	Manual palpation	5 min
Carvalho and Barroso (2019b)	85% 1RM knee extension to failure	Acute IPC	Alternate thighs (unilateral)	4 × 5 min	250 mm Hg	None	10 mm Hg	NA	30 min
Carvalho and Barroso (2019a)	Isometric knee extension MVC test	Acute IPC	Alternate thighs (unilateral)	4 × 5 min	250 mm Hg	None	10 mm Hg	NA	NA
Cheung et al. (2020)	Incremental exercise test (Cycling)	Acute IPC	Alternate thighs (unilateral)	4 × 5 min	Individual AOP	40 min supine passive rest	Therapeutic ultrasound	Personal Tourniquet System	Immediately
Clevidence et al. (2012)	Incremental exercise test (Cycling)	Acute IPC	Alternate thighs (unilateral)	3 × 5 min	220 mm Hg	30 min supine passive rest	None	NA	5 min
Cocking et al. (2017)	1 h TT (Cycling)	Acute IPC and RIPC	Alternate arms and thighs (bilateral)	4 × 5 min	220 mm Hg	20 mm Hg	None	NA	Immediately
Cocking et al. (2018b)	375 kJ TT (Cycling)	Acute IPC + RIPC	Thigh (bilateral) Left thigh (unilateral) Arm (bilateral) Thigh (bilateral)	4 × 5 min 4 × 5 min 4 × 5 min 8 × 5 min	220 mm Hg	20 mm Hg	None	NA	20 min
Crisafulli et al. (2011)	Incremental exercise test + TTE (Cycling)	Acute IPC	Thigh (bilateral)	3 × 5 min	50 mm Hg > SBP	Reference test	None	NA	5 min
Cruz et al. (2015)	TTE at 100% PPO (Cycling)	Acute IPC	Thigh (bilateral)	4 × 5 min	220 mm Hg	20 mm Hg	None	NA	90 min
Cruz et al. (2016)	60 s Sprint (Cycling)	Acute IPC	Thigh (bilateral)	4 × 5 min	220 mm Hg	20 mm Hg	None	NA	33 min
da Mota et al. (2019)	2 × 5 km TT (Cycling)	Acute IPC	Thigh (bilateral)	3 × 5 min	220 mm Hg	None	20 mm Hg	NA	25 min
De Groot et al. (2010)	Incremental exercise test (Cycling)	Acute IPC	Thigh (bilateral)	3 × 5 min	220 mm Hg	No occlusion	None	NA	5 min
El Messaoudi et al. (2013)	70 min at 85% HRmax + TTE at 95% HRmax (Cycling)	Acute RIPC	Forearm (bilateral)	3 × 5 min	200 mm Hg	Reference test	None	NA	Immediately
Ferreira et al. (2016)	6 × 50 m Sprint (Swimming)	Acute IPC	Thigh (bilateral)	3 × 5 min	220 mm Hg	10 mm Hg	3 × 1 min at 220 mm Hg	NA	10 min
Foster et al. (2011)	100 kJ TT (Cycling)	Acute IPC	Thigh (unilateral)	4 × 5 min	20 mm Hg > SBP	No occlusion	None	Pulse oximeter	90 min
Franz et al. (2018)	3 × 10 Biceps curls at 80% 1RM	Acute IPC	Arm (bilateral)	3 × 5 min	200 mm Hg	No occlusion	None	NA	5 min
Garcia et al. (2017)	Agility *T*-Test + countermovement jump + 30s jump test	Acute IPC	Alternate thighs (unilateral)	3 × 5 min + 2 min reperfusion	220 mm Hg	21 min seated passive rest	None	NA	1 min
Gibson et al. (2013)	3 × 30 m Sprint (Running)	Acute IPC	Alternate thighs (unilateral)	3 × 5 min	220 mm Hg	No occlusion	50 mm Hg	NA	5 min
Gibson et al. (2015)	5 × 6 s Sprint (Cycling)	Acute IPC	Alternate thighs (unilateral)	3 × 5 min	220 mm Hg	No occlusion	50 mm Hg	NA	5 min
Griffin et al. (2018)	3 min sprint (Cycling)	Acute	Thigh (bilateral)	4 × 5 min	220 mm Hg	20 mm Hg	None	NA	Immediately
Griffin et al. (2019)	Repeated sprint (Running)	Acute IPC + RIPC	Arm (bilateral) Thigh (bilateral)	4 × 5 min 4 × 5 min	220 mm Hg 220 mm Hg	20 mm Hg	None	NA	15 min
Halley et al. (2018)	Isometric knee extension 2 min MVC	Acute IPC	Alternate thighs (unilateral)	3 × 5 min	220 mm Hg	30 min passive rest	20 mm Hg	NA	20 min
Halley et al. (2019)	6 × 11 MVC knee extension in normoxia and hypoxia	Acute IPC	Alternate thighs (unilateral)	3 × 5 min	220 mm Hg	None	20 mm Hg	NA	10 min
Hittinger et al. (2014)	Incremental exercise test at sea level and simulated altitude (Cycling)	Acute IPC	Thigh (bilateral)	4 × 5 min	10 mm Hg > SBP	No occlusion	None	Manual palpation	45 min
Huang et al. (2020)	Isokinetic knee extension/flexion strength and endurance	Acute IPC	Thigh (bilateral)	3 × 5 min	50 mm Hg > SBP	10 mm Hg	None	NA	5 min
James et al. (2016)	Incremental exercise test in 32°C (Running)	Acute IPC	Thigh (bilateral)	4 × 5 min	220 mm Hg	None	50 mm Hg	NA	5 min
Jean-St-Michel et al. (2011)	7 × 200 m submaximal + 100/200 m TT (Swimming)	Acute RIPC	Arm (unilateral)	4 × 5 min	15 mm Hg > SBP	10 mm Hg	None	NA	45 min
Jeffries et al. (2019)	Submaximal + incremental exercise test (Cycling)	Repeat IPC	Thigh (bilateral)	4 × 5 min for 7 days	220 mm Hg	20 mm Hg	None	Doppler	72 h
Kaur et al. (2017)	Incremental submaximal (Running)	Acute IPC	Thigh (bilateral)	3 × 5 min	220 mm Hg	20 mm Hg	None	NA	15 min
Kido et al. (2015)	Work-To-Work test (Cycling)	Acute IPC	Thigh (bilateral)	3 × 5 min	>300 mm Hg	30 min passive rest	None	NIRS	5 min
Kilding et al. (2018)	Incremental exercise test + 4 km TT (Cycling)	Acute IPC	Thigh (bilateral)	4 × 5 min	200 mm Hg	30 mm Hg < DBP	None	NA	5 min
Kjeld et al. (2014)	Static/dynamic apnea + 1000 m TT (Rowing)	Acute RIPC	Forearm (unilateral)	4 × 5	40 mm Hg > SBP	No occlusion	None	NA	30 min
Kraus et al. (2015)	4 × 30 s Wingate Anaerobic Test	Acute RIPC	Left arm (unilateral) Arm (bilateral)	4 × 5 min	NA	10 mm Hg	None	NA	15 min
Lalonde and Curnier (2015)	6 s Sprint + Wingate Anaerobic Test	Acute RIPC	Right arm (unilateral)	4 × 5 min	50 mm Hg > SBP	10 mm Hg	None	NA	NA
Libonati et al. (1998)	15 × isometric wrist flexion MVC	Acute IPC	Forearm (unilateral)	1 × 2 min + 10 s reperfusion	200 mm Hg	No occlusion	None	NA	Immediately
Lindsay et al. (2017)	Simulated Keirin test (Cycling)	Repeat IPC	Alternate thighs (unilateral)	4 × 5 min for 7 days	220 mm Hg	20 mm Hg	None	Pulse oximeter	24 h
Lisbôa et al. (2017)	3 × 50 m TT (Swimming)	Acute IPC and RIPC	Thigh (bilateral) Arm (bilateral)	4 × 5 min 4 × 5 min	220 mm Hg 180 mm Hg	20 mm Hg	None	NA	1, 2, and 8 h
Lopes et al. (2018)	Repeated shuttle sprint (Running)	Acute IPC	Alternate thighs (unilateral)	3 × 5 min	220 mm Hg	10 mm Hg	None	Doppler	10 min
Marocolo et al. (2016b)	12RM leg extension	Acute IPC	Alternate thighs (unilateral)	4 × 5 min	220 mm Hg	40 min seated passive rest	20 mm Hg	Auscultation	8 min
Marocolo et al. (2015a)	100 m TT (Swimming)	Acute IPC	Alternate arms (unilateral)	4 × 5 min	220 mm Hg	40 min passive rest	20 mm Hg	NA	5 min
Marocolo et al. (2016a)	12RM elbow flexion	Acute IPC + RIPC	Alternate arms or thighs (unilateral)	4 × 5 min	220 mm Hg	Reference test	20 mm Hg	Auscultation	6 min
Marocolo et al. (2017)	Incremental shuttle run	Acute IPC	Alternate thighs (unilateral)	4 × 5 min	220 mm Hg	40 min seated passive rest	20 mm Hg	Auscultation	6 min
McIlvenna et al. (2019)	Incremental exercise test + 16.1 km TT (Cycling)	Acute IPC	Thigh (bilateral)	3 × 5 min	180 mm Hg	Reference test	None	Doppler	10 min
Mieszkowski et al. (2020)	Marathon running	Repeat IPC	Thigh (bilateral)	4 × 5 min for 10 days	220 mm Hg	20 mm Hg	None	Doppler	24 h
Mota et al. (2020)	3 min sprint (Arm cycling)	Acute IPC	Arm (bilateral)	3 × 3 min + 2 min reperfusion	50 mm Hg > SBP	20 mm Hg	None	NA	10 min
Paixão et al. (2014)	3 × Wingate Anaerobic Test	Acute IPC	Alternate thighs (unilateral)	4 × 5 min	250 mm Hg	20 mm Hg	None	NA	12 min
Paradis-Deschênes et al. (2018)	5 km TT at Low + Moderate + High Altitude (Cycling)	Acute IPC	Alternate thighs (unilateral)	3 × 5 min	220 mm Hg	None	20 mm Hg	NIRS	25 min
Paradis-Deschênes et al. (2020b)	Repeat 5 km TT (Cycling)	Acute IPC PE	Alternate thighs (unilateral)	3 × 5 min	220 mm Hg	No occlusion	None	NA	15 min before 2nd TT
Paradis-Deschênes et al. (2016)	5 × 5 MVC knee extension	Acute IPC	Right thigh (unilateral)	3 × 5 min	200 mm Hg	None	20 mm Hg	NIRS	18 min
Paradis-Deschênes et al. (2017)	5 × 5 MVC knee extension	Acute IPC	Right thigh (unilateral)	3 × 5 min	200 mm Hg	None	20 mm Hg	NIRS	19 min
Paradis-Deschênes et al. (2020a)	Wingate Anaerobic Test + 5 km TT + incremental exercise test (cycling)	Repeat IPC	Alternate thighs (unilateral)	3 × 5 min 2 × /week for 4 weeks	220 mm Hg	None	20 mm Hg	NA	pre-mid- and post-training
Patterson et al. (2015)	12 × 6 s repeated sprinting (Cycling)	Acute IPC	Thigh (bilateral)	4 × 5 min	220 mm Hg	20 mm Hg	None	NA	30 min
Paull and Van Guilder (2019)	Supramaximal TTE (Running)	Acute RIPC	Right arm (unilateral)	4 × 5 min	220 mm Hg	20 mm Hg	None	NA	15 min
Pereira et al. (2020)	Isometric plantarflexion TTE at 20% MVC	Acute IPC and RIPC	Non-dominant thigh/arm (unilateral)	3 × 5 min	225 mm Hg	30 min seated passive rest	225 mm Hg for 1 min	NA	Immediately
Richard and Billaut (2018)	1000 m TT (Speed Skating)	Acute RIPC	Alternate arms (unilateral)	3 × 5 min	30 mm Hg > SBP	10 mm Hg	None	NA	90 min
Sabino-Carvalho et al. (2017)	Discontinuous incremental exercise (Running)	Acute IPC	Alternate thighs (unilateral)	4 × 5 min	220 mm Hg	40 min supine passive rest	Therapeutic ultrasound	Doppler	10 min
Seeger et al. (2017)	5k TT (Running)	Acute IPC	Thigh (bilateral)	4 × 5 min	220 mm Hg	20 mm Hg	None	NA	1 and 24 h
Slysz and Burr (2018)	Wingate Anaerobic Test + incremental exercise test (Cycling)	Acute IPC	Thigh (bilateral)	3 × 5 min 3 × 5 min + walk 3 × 5 min + EMS	220 mm Hg	30 min seated passive rest	None	NIRS	10 min
Slysz et al. (2019)	5 km TT (Cycling)	Acute IPC	Thigh (bilateral)	3 × 5 min 3 × 5 min × 2 3 × 5 min × 3	Individual AOP	No occlusion No occlusion No occlusion	None	Personal Tourniquet System	15 min 24 h and 15 min 48 h 24 h and 15 min
Slysz and Burr (2018)	Incremental exercise test + 1,000 m TT (Running)	Repeat IPC	Right thigh (unilateral)	3 × 5 min 6 × /week for 8 weeks	Individual AOP	No occlusion	None	Personal Tourniquet System	48 h to 7 days after last IPC
Surkar et al. (2020)	Wrist extensor 1RM + EMG (Strength training)	Repeat IPC	Dominant arm (unilateral)	5 × 5 min for 6 days	20 mm Hg > SBP	10 mm Hg < DBP	None	NA	7 days after final training day
Tanaka et al. (2016)	Isometric knee extension TTE at 20% MVC	Acute IPC	Right thigh (unilateral)	3 × 5 min	>300 mm Hg	30 min passive rest	None	NA	5 min
Telles et al. (2020)	3 × 80% 1RM bench press/leg press TTE	Acute IPC	Alternate thighs (unilateral)	4 × 5 min	220 mm Hg	20 mm Hg	None	Calculation	5 min
Thompson et al. (2018)	10 m + 20 m sprint (Running)	Acute IPC	Right thigh (unilateral)	3 × 5 min	220 mm Hg	No occlusion	20 mm Hg	NA	15 min
Tocco et al. (2015)	5 km TT (Running)	Acute IPC	Thigh (bilateral)	3 × 5 min	50 mm Hg > SBP	Reference test	10 mm Hg < DBP	NA	5 min
Tomschi et al. (2018)	Incremental exercise test (Cycling)	Acute RIPC	Right arm (unilateral)	4 × 5 min	200 mm Hg	10 mm Hg	120 mm Hg	Pulse oximeter	NA
Turnes et al. (2018)	2000 m TT (Rowing Ergometer)	Acute IPC	Alternate thighs (unilateral)	3 × 5 min 3 × 10 min	220 mm Hg	None	20 mm Hg	NIRS	30 min
Valenzuela et al. (2019)	Force-velocity/rep-to-failure at 60% 1RM (Bench Press)	Acute IPC	Arm (unilateral)	3 × 5 min	220 mm Hg	10 mm Hg	None	NA	40 min
Wiggins et al. (2019)	5 km TT in normoxia and hypoxia (Cycling)	Acute IPC	Alternate thighs (unilateral)	4 × 5 min	220 mm Hg	None	20 mm Hg	NA	10 min
Williams et al. (2018)	100 m TT (Swimming)	Acute IPC	Thigh (bilateral)	4 × 5 min	Individual AOP	15 mm Hg	None	Calculation	2 and 24 h
Zinner et al. (2017)	16 × 30 m multidirectional sprint (Running)	Acute IPC + RIPC	Thigh (bilateral) Arm (bilateral)	3 × 5 min	240 mm Hg 185 mm Hg	20 mm Hg	None	NA	40 min

*AOP, arterial occlusion pressure; CON, control condition; DBP, diastolic blood pressure; EMG, electromyography; EMS, electrical muscle stimulation; GET, gas exchange threshold; HRmax, maximal heart rate; IPC, ischemic preconditioning; IPC and RIPC, both local and remote ischemic preconditioning applied simultaneously; IPC + RIPC, local and remote ischemic preconditioning applied on separate visits; MVC, maximal voluntary contraction; NA, not available; NIRS, near infrared spectroscopy; PE, post-exercise; PLA, placebo condition; PPO, peak power output; RIPC, remote ischemic preconditioning; SBP, systolic blood pressure; TTE, time to exhaustion; TT, time trial; 1RM, one repetition maximum; 12RM, twelve repetition maximum. The distinction between low-pressure CON and low-pressure PLA treatments was made based on whether the original investigators attempted to blind participants to the treatment through deception or nocebo techniques. Investigations that have utilized low-pressure treatments but have not attempted to blind participants to the different treatment pressures are defined as control treatments in this table*.

A consequence of the methodological variations reported in the literature relate to the identification of the physiological mediators of the beneficial effects of IPC. Information gleaned from clinical experiments suggests that the protective effects of IPC operate through neuronal, humoral, and systemic pathways (Marongiu and Crisafulli, 2014). Others propose that IPC promotes reactive hyperemia mediated by nitric oxide production (Singh et al., 2017), phosphocreatine resynthesis (Andreas et al., 2011), peripheral hemodynamics (Paradis-Deschênes et al., 2016; Cocking et al., 2018a; Halley et al., 2018), and skeletal muscle oxygen uptake (Andreas et al., 2011; Paradis-Deschênes et al., 2016). Such physiological responses have a theoretical basis that would support enhanced exercise performance capacity, but the heterogeneous methodologies described in Table 1 and the varying positive response rates to IPC reported in the literature, when considered together, challenge the confident identification of physiological mediators of performance changes associated with IPC. This knowledge gap similarly challenges knowledge transfer and application, specifically being able to confidently predict whether an IPC intervention is likely to result in ergogenic effects.

## Methodological Variations Contributing To Heterogeneous Responses

### IPC Protocol

There appears to be no scientific consensus regarding the IPC protocol most likely to elicit an ergogenic effect. Table 1 demonstrates that there are substantial variations in the chosen IPC protocols. For example, the number of ischemic cycles used has ranged from one to eight cycles (Libonati et al., 1998; Cocking et al., 2018b), and the durations of ischemia have ranged from 2 to 10 min (Libonati et al., 1998; Andreas et al., 2011). Most, but not all investigations allow for a duration for reperfusion between ischemic intervals that is equal to the duration of the ischemic interval, however, some studies have utilized longer (Andreas et al., 2011) or shorter reperfusion intervals (Libonati et al., 1998; Garcia et al., 2017; Mota et al., 2020). The most common IPC protocols have been four (47% of studies) or three (42% of studies) cycles of 5 min ischemia-reperfusion. Most reports to date have conducted their exercise testing acutely on the same day as the IPC stimulus. However, a minority of studies have conducted exercise testing after repeated daily IPC exposure ranging from 7 days to 6 weeks (Banks et al., 2016; Jeffries et al., 2019; Slysz and Burr, 2019; Mieszkowski et al., 2020; Paradis-Deschênes et al., 2020a; Surkar et al., 2020). There also is substantial variation in whether IPC is administered unilaterally or bilaterally to the limb(s), which may influence the magnitude of response. Such heterogeneous methods confound the comparison of results among various studies.

Understanding whether or not there is an optimal dose-response for IPC would seem to be integral to clarifying the probability of beneficial effects on a specified type, duration, or intensity of exercise performance. Surprisingly few studies have compared and contrasted the effects of different IPC protocols on exercise performance outcomes. Turnes et al. (2018) compared three cycles of 5 min vs. 10 min IPC but did not find a benefit of either treatment on rowing ergometry performance. De Groot et al. (2010) demonstrated that fewer than three cycles of IPC had negligible effects on athletic performance whereas Cocking et al. (2018b) did not find additional benefits of IPC when comparing eight cycles to four cycles of 5 min IPC. These authors also found that a unilateral IPC approach resulted in significantly slower cycling time trial performance compared to bilateral IPC which suggests that the IPC response is contingent on the total tissue area under ischemia. It has been suggested that the protective effects of IPC might require achieving a physiological disruption that is reflected in the accumulation of metabolites above a threshold, eliciting synergistic effects that propagate the IPC response (Cohen et al., 2000; Marongiu and Crisafulli, 2014). Such a theory implies that the total volume of tissue and/or duration of ischemia-reperfusion may be important to meet this metabolite threshold. Additional effects of IPC may also be dependent on the time or frequency of the IPC stimulus. For example, the degree of RIPC induced conduit artery vasodilation has been shown to be dependent on the number of ischemic cycles (Enko et al., 2011), and the hyperemic response to tourniquet occlusion is dependent on the duration of ischemia (Johnson et al., 1976).

Taken together, this evidence suggests that the probability and magnitude of an ergogenic response to IPC is a function of an “entourage” effect that is a cumulative consequence of some combination of duration, frequency, and/or tissue volume dependent stimulus. Until further research clarifies the magnitude of the individual and entourage contributions, the evidence in Table 1 leads to our recommendation that IPC protocols should entail at least three cycles of 5 min ischemia-reperfusion, and that IPC should be applied bilaterally based on the observations of increased performance when occluding both limbs compared to one (Cocking et al., 2018b).

### Absolute vs. Relative Tourniquet Pressures

Perhaps the most pervasive source of methodological variability within the IPC exercise research is the tourniquet pressures chosen to elicit IPC. Table 1 indicates that pressures have ranged from 10 mm Hg above systolic blood pressure (Hittinger et al., 2014) to those in excess of 300 mm Hg (Kido et al., 2015; Tanaka et al., 2016). The literatures suggests that absolute tourniquet pressures in the range of 200–250 mm Hg have been commonly used regardless of body size. The use of these absolute pressures appear to be largely based on favorable responses reported in previous publications (De Groot et al., 2010; Ferreira et al., 2016; Garcia et al., 2017). Yet, it is surprising given how widely variable arterial occlusion pressures (AOP) are among individuals, not to mention the potential for vast differences in the physical characteristics of the tourniquet. Lack of consideration of individual AOP may augment safety risks as higher tourniquet pressures are associated with greater risks of tourniquet-related injuries (Ochoa et al., 1972; Murphy et al., 2005). Thus, to minimize the risk and maximize the outcome, the minimum pressure required to prevent the flow of arterial blood into the limb should be established whenever possible in IPC interventions.

Past studies have demonstrated individual variations in lower limb AOP ranging from 100 mm Hg to above 300 mm Hg (Loenneke et al., 2012, 2013). These differences can be attributed to a multitude of factors including limb circumference, blood pressure, and biological sex (Crenshaw et al., 1988; Graham et al., 1993; Loenneke et al., 2015; Brown et al., 2018). Limbs with larger tissue masses require higher tourniquet pressures to achieve arterial occlusion (Graham et al., 1993; Loenneke et al., 2015). This would seem to constitute an important consideration for determining the tourniquet pressures to use when applying IPC to male vs. female or trained vs. untrained individuals. Body position also affects AOP as demonstrated by Sieljacks et al. (2018) who reported the need for larger tourniquet pressures to occlude leg blood flow in the seated position compared with the supine position. The dimensions of the tourniquet itself should also be considered as wider cuffs with evenly distributed pressure gradients require lower external pressures to sufficiently occlude blood flow compared to narrow cuffs (Crenshaw et al., 1988; Loenneke et al., 2012; Brown et al., 2018). In light of the preceding discussion, we contend that it is likely an erroneous assumption that all participants will experience a similar level of arterial constriction with the application of the same tourniquet pressure.

Despite these important considerations, two-thirds of the IPC exercise studies found in Table 1 have failed to report any confirmation of AOP. Only five of the 81 studies have prescribed pressures relative to individual AOP. Therefore, it cannot be concluded that all subjects in these analyses attained similar and/or sufficient degrees of ischemia to stimulate a response. We posit that this may contribute to the equivocal findings reported in the literature and/or the variation in response rates within the same study. We recommend that the identification of individual minimal AOP is a key parameter that should be monitored continuously during the IPC intervals to ensure the absence of persistent blood flow, reported, and used in future IPC research.

### Remote or Local IPC

It seems reasonable to speculate that the location of the IPC stimulus relative to the musculature that will be activated during subsequent exercise might impact the success of the intervention. Most of the studies reviewed in Table 1 have utilized local IPC applied in close proximity to the muscle groups that will be exercising, with mixed success in finding an ergogenic effect. Eighteen of the 81 studies examined RIPC on exercise performance. Of those reports, few have directly compared the effects of IPC and RIPC on performance outcomes. Based on the limited available evidence, there does not appear to be a difference in performance outcomes between IPC and RIPC interventions; three studies have shown no effect of either technique on performance (Marocolo et al., 2016a; Zinner et al., 2017; Griffin et al., 2019) whereas one showed similar improvements for both on cycling performance (Cocking et al., 2018b).

The evidence that central humoral, neural, and systemic ischemic protection mediate IPC benefits suggests that the location of the application of IPC may not have an optimal site of application. Alternatively, muscle deoxygenation responses during exercise preceded by local (Paradis-Deschênes et al., 2016, 2020a), but not always remote IPC (Barbosa et al., 2015), may suggest that the IPC stimulus is optimized when administered in close proximity to the exercising tissues. Without a comprehensive understanding of the mechanisms most likely to elicit an ergogenic response, it is difficult to determine if there are advantages favoring remote or local IPC in terms of the probability of an ergogenic effect. More focused research is definitely required about this issue.

### Time Between IPC Application and Exercise

It remains unclear what the optimal duration is between completion of IPC application and the start of exercise. This is surprising because it also seems to be a relatively fundamental assumption that such an optimal duration should exist. The void of information likely also contributes to the varied responses reported in the literature. Table 1 demonstrates the wide range of durations between IPC and exercise testing used throughout the literature. Most studies (~70%) report beginning exercise within the first 30 min after IPC, however several studies reported commencing exercise between 30 min to 72 h after IPC depending on the objectives of the investigation. We conclude that the chosen interval between IPC and exercise appears to be largely arbitrary as few investigations have sought to compare the effects of different durations on performance.

An improved understanding of the mediators of ischemic protection associated with IPC, and how they may mediate ergogenic effects is integral to identifying the optimal time-to-exercise after IPC. For example, the hyperemic response following IPC is not likely to persist beyond the initial several minutes following IPC. Moreover, it is not clear how long after IPC that increases in exercising muscle O_2_ extraction persist. Based on these phenomena alone, one may conclude that immediate exercise is favorable for eliciting an ergogenic response through enhanced convective O_2_ delivery and diffusion at the muscle capillary interface. Nonetheless, the contribution of the protective effects against ischemia from IPC are equivocal when it comes to their application to exercise. Therefore, we do not know if the mediators of the purported ergogenic response occur relatively instantaneous or if there is a latent response.

For purposes of comparison, in clinical studies IPC is known to have two windows of protection characterized by substantial reductions in tissue infarct size during sustained ischemia. The first window of protection lasts ~3 h after application of IPC, followed by a 12–24 h period without protective effects. Then, a second window of protection occurs that lasts upwards of 72–90 h from the IPC stimulus (Pagliaro et al., 2001; Marongiu and Crisafulli, 2014). The persistence of these protective windows may help explain why IPC has been shown to influence performance 8–24 h after application (Lindsay et al., 2017; Lisbôa et al., 2017). Still, we contend that the optimal duration between IPC and exercise has not been sufficiently explored to permit confidence in drawing conclusions. Seeger et al. (2017) compared 5 km treadmill running tests 5 min and 24 h after IPC but found no effect of either duration on performance. Lisbôa et al. (2017) examined time-dependent effects of IPC on 50 m swim performance at 1 h, 2 h, and 8 h following IPC. They did not find improved performance within 1 h of IPC despite showing clear benefits in the 2 h and 8 h trials which may point to a delayed onset of ergogenic effects. This, however, does not explain the bulk of studies that have found performance enhancement within the first hour after IPC (Jean-St-Michel et al., 2011; Marocolo et al., 2015a, 2016a,b; Cruz et al., 2016; Paradis-Deschênes et al., 2016, 2017, 2018).

Therefore, although most studies have conducted their exercise testing within the first 30 min of IPC, we can find no evidence of the determination of an optimal time between IPC and exercise. The establishment of guidelines for IPC protocols will be challenging at best and lack empirical support until the potential time-dependent effects of IPC and exercise performance are clarified.

### Is IPC a Placebo Effect?

Due to the obvious differences in the subjective sensation of limb arterial occlusion vs. a low-pressure sham condition, it is inherently challenging to incorporate a proper placebo treatment into IPC exercise research. This presents challenges with adequately blinding participants to the treatments being studied and more than likely introduce biases into an investigation. Given these challenges, it is perhaps unsurprising that many previous investigations have forgone the incorporation of placebo treatments into their studies (Marocolo et al., 2015b, 2019). In our review of the literature, we have found that only 33% of IPC exercise studies have incorporated a placebo intervention in their study design (Table 1). For further clarity, we have defined a placebo as an intervention in which participants were led to believe that the sham treatment would have similar outcomes to the IPC treatment. Studies that used low-pressure sham conditions without deception or placebo-nocebo expectations have been presented as control conditions in our table.

The disconcerting low number of placebo-controlled IPC studies was highlighted previously in a letter to the editor in response to a systematic review by Incognito et al. (2016); the letter reported that 50% of the studies in the review showing performance benefits did not include a placebo in their study designs (da Mota and Marocolo, 2016). Only 24% of the investigations that reported performance improvements following IPC in the Incognito et al. review controlled for potential placebo effects.

There is additional earlier evidence suggesting that performance enhancement following IPC may be, at least partly, a placebo effect (Marocolo et al., 2015a, 2016a,b; Sabino-Carvalho et al., 2017). Marocolo et al. (2015a) found no difference between IPC and a low-pressure placebo after discovering significant improvements in 100 m swimming following IPC compared to a control. Separate reports from the same group found that both IPC and placebo conditions increased the maximal number of repetitions performed during elbow flexion and knee extension exercise compared to baseline testing (Marocolo et al., 2016a,b). Whether these studies indicate a placebo effect or potentially an experimental learning or order effect is difficult to ascertain from the published results. Similarly, Sabino-Carvalho et al. (2017) reported improved time-to-exhaustion after IPC with no differences between IPC and a therapeutic ultrasound sham condition, and no difference between conditions in the measured physiological variables.

There are reports that run counter to the assumption of a placebo effect to IPC. For example, Ferreira et al. (2016) conducted a placebo-nocebo controlled investigation on repeated 50 m swimming performance. Despite 73% of participants expecting their performance to improve following the sham condition, only IPC improved swimming performance relative to the control. Similarly, Cheung et al. (2020) reported improved cycling time-to-exhaustion following IPC despite participants reporting negative performance expectations after IPC and positive performance expectations after their sham.

Thus, it remains unclear whether the purported ergogenic effects of IPC are a placebo effect or a physiologically evoked and mediated response to the IPC intervention. The current lack of placebo incorporation in IPC exercise studies is a trend that should be rectified in future investigations. This will require effective techniques aimed to blind participants to the treatment of interest when conducting IPC research. This may be achieved through the incorporation of placebo- nocebo controls or deception when feasible.

## Conclusion

This review is intended to raise awareness of the wide range and inconsistencies in methodologies that confound interpretation of the literature about the efficacy of IPC as an ergogenic aid. We highlight the wide-ranging variations in chosen IPC protocols, tourniquet pressures, location of the IPC stimulus, and the time between treatment and exercise as potential sources for the discordant response rates. Furthermore, the importance of incorporating quality placebo treatments into future investigations should not be overlooked. More consistent methodologies are critical to being able to eventually develop evidence-based guidelines for IPC applications designed to enhance athletic performance.

## Author Contributions

LO'B and IJ contributed to the writing, reading of this manuscript, contributed to critical revisions of the manuscript, reviewed and approved the final version of this manuscript, and agree to be accountable for the accuracy of the information presented. Collection and interpretation of the literature review was undertaken by LO'B. Drafting and formatting of the article was undertaken by LO'B and IJ. All authors contributed to the article and approved the submitted version.

## Conflict of Interest

The authors declare that the research was conducted in the absence of any commercial or financial relationships that could be construed as a potential conflict of interest.
